# Supplementary material for: Are carbon emissions trading and green financial instruments synergistic? -Comprehensive quantitative research based on content analysis
Source: PLoS One. 2024 Mar 7;19(3):e0298601. doi: 10.1371/journal.pone.0298601 (PMC10919601; doi:10.1371/journal.pone.0298601)
Supplement: S1 Data — (ZIP) [file pone.0298601.s001.zip › Data/地级市Z_F工作报告.zip!/║╙▒▒/╒┼╝╥┐┌╩╨/└┤╫╘╣1⁄2╓┌║┼í╛┬φ┐╦╩2╛▌═°í┐_2015.docx]

各位代表：

现在，我代表张家口市人民政府向大会作工作报告，请予审议，并请市政协委员和列席会议的同志提出意见。

一、2014年主要工作回顾

2014年，面对宏观经济复杂多变的形势，在市委的正确领导下，在市人大、市政协的监督和支持下，紧紧团结依靠全市人民，立足习总书记对我市建设京津冀水源涵养功能区的定位，抢抓京张联合申奥和京津冀协同发展两大历史机遇，以发展奥运、园区、生态、县域“四型经济”为抓手，凝心聚力，创新奋进，经济社会发展保持了良好态势。初步核算，全市地区生产总值完成1358.5亿元，增长5.2%。固定资产投资完成1402亿元，增长10.2%。社会消费品零售总额完成556.5亿元，增长12.5%。利用外资3.2亿美元，增长17.5%；外贸进出口总值5.2亿美元，增长34.4%。全部财政收入完成230.6亿元，增长2.6%；地方公共财政预算收入完成125.8亿元，增长6.2%。规模以上工业增加值完成428.1亿元，增长4.9%。民营经济增加值占生产总值的比重达到58%。单位GDP能耗预计降低4.9%左右。全市新增就业5.34万人；城乡居民人均可支配收入分别达到21651元和7462元，分别增长10.2%和13.3%，增速均居全省第一；居民消费价格指数上涨1.1%，低于全国、全省平均水平。

(一)申奥工作顺利推进。成功入围2022年冬奥会候选城市。高质量完成了调查问卷、视频质询、申办报告等各项工作。赛场规划已经完成，云州水库调水、雪场配套设施等重点工程已完成年度任务。成功举办了国际滑雪节、冰雪嘉年华等活动。经国际奥委会单项组织考察，我市的5个比赛场地全部通过认证。迎接国际奥委会评估委员会考察工作全面展开。实施了迎宾廊道、雪场达10万亩的绿化工程。同时，以道路为核心的赛场基础设施建设正在积极进行，京张高铁八达岭段可研获国家发改委批准。

(二)发展质量有新的提高。工业加快转型升级。工业固定资产投资完成566.5亿元，增长32.8%，增幅全省第一；实施技改项目350项，总投资274亿元，增长9.7%；新增规上工业企业98家。出台了《支持工业转型发展八项措施》，进一步提升企业技术装备和创新能力，张煤机、北大青鸟等一批企业实现转型发展，我市被工信部列入“全国工业绿色转型发展试点”城市。新增11家省“两化”融合重点企业，长城葡萄酒公司被评为国家级“两化”深度融合示范企业。张煤机被评为国家火炬计划重点高新技术企业。全市新增省级科技企业孵化器1家、重点实验室2家。农业主导产业持续壮大。规模经营步伐进一步加快，新流转土地49.9万亩，各类农业园区达到309个。克服旱情较重等不利影响，农业主导产品产量稳步增长，畜牧、葡萄、蔬菜、马铃薯占农业总产值80%以上。“张杂谷”市外推广100万亩，并走出国门，赵治海杂交谷子科研团队获省科学技术突出贡献奖。农业全产业链企业逐步增多，市级以上龙头企业发展到375家，产业化经营率达到63%。现代服务业发展势头良好。借助奥运效应，冰雪游、草原游等特色旅游日益火爆，全年接待国内外游客3318万人次、增长20.3%，旅游总收入达到237.6亿元、增长28.5%。崇礼滑雪接待游客数量再创历史新高，较去年同期增长了28.6%。金融服务业稳步提升，支持企业在多层次资本市场直接融资，嶂谷农业、华凌建材、三祖庄园等7家企业成功挂牌上市。电子商务加快发展，建成了沙城葡萄酒、坝上农产品等4家电子商务平台，成为省农村电子商务“农村火种计划”唯一试点城市。第三产业占比达到40.5%，比上年提高0.5个百分点。新型城镇化有序推进。城市管理的智能化、精细化水平不断提升，交通拥堵、狗患等一些群众反映强烈的问题得到有效治理。县城的供水、燃气、集中供热普及率分别达到100%、76.4%和77%。350个省级农村面貌改造提升重点村累计投入10.4亿元，村庄面貌得到明显改善。怀来、张北两县成功创建“国家园林县城”，涿鹿、赤城、崇礼、怀安、万全5个县被评为“省级园林县城”。万全、张北两县荣获2013—2014年度河北省人居环境进步奖，怀来县获得人居环境奖，张北县被确定为“国家级新型城镇化试点”。

(三)发展后劲显著提升。实施了重大项目攻坚。200项重点建设项目完成投资596.6亿元，超过年计划29个百分点，其中16个省级重点项目完成投资91.8亿元，占年计划的100.9%，在全省重点项目建设观摩暨调度会上综合评价位列第一。生产性项目占到了75.5%，同比增加18.5个百分点。旗帜乳品项目当年开工、当年建成投产，创造了张家口速度。蔚县电厂经20多年的努力，正式开工建设。云计算、中法生态产业园等投资上百亿元的7个项目顺利推进。高新技术产业项目有了突破性进展，通过“基金+项目”的平台经济模式，从上海张江科技园区、美国硅谷等地，引进了43个拥有四代技术的高新技术项目，其中矽光光电推出的“新一代三维LED”项目荣获第三届中国创新创业大赛新能源行业总决赛第一名。园区建设稳步推进。全市省级园区达到15个，其中省级高新技术产业开发区4家。入园企业881家，实现销售收入478亿元。张江张家口高新技术产业园、中瑞中小企业国际合作园成功挂牌并加速建设，怀来航天产业园、中关村——张北云计算产业园、北方硅谷高科新城等专业园区快速起步，西山产业集聚区被工信部评为装备制造业国家新型工业化产业示范基地，中煤机装备产业园获得中国建筑最高奖——鲁班奖。园区管理体制正在逐步理顺，新区和经开区得到了整合。生态环境进一步改善。全年完成绿化131.8万亩，森林覆盖率达36%，成功创建全省首个“国家森林城市”，并入围国家第一批生态文明先行示范区，山体绿化建设项目荣获中国人居环境范例奖。治理水土流失面积241.5平方公里，实施高效节水灌溉24万亩。大气污染治理超额完成省下达的各项任务，取缔煤炭经营企业346家，淘汰燃煤锅炉394台、黄标车3.5万辆。化学需氧量、二氧化硫、氨氮、氮氧化物分别削减1.2%、1.1%、2.0%和12.5%，PM2.5浓度下降5.8%，完成省下达任务。全年空气质量优良天数315天，同比增加30天，空气质量综合指数位列长江以北监测城市第一。交通基础设施持续提升。张承二期、京新三期等高速公路加快建设，全市高速公路通车总里程达到825.5公里，居全国设区市前列。张唐、张呼铁路进展顺利，张大、蓝张铁路获得国家发改委批复。

(四)发展活力进一步增强。大力简政放权。市本级对行政许可、非行政许可审批和行政监管“三类事项”进行了彻底清理，分五批精简市级行政审批事项430项，涉及行政许可182项、非行政许可59项、行政监管189项，削减比例达65%。对于确需保留的233项审批项目列出清单，在市政府门户网站和审批大厅进行了公布。“四级平台、三个代办”工作有序推进。全面推行“涉企一口收费制”，所有涉企行政事业性收费项目全部进入市、县政务服务中心统一征收。启动了宣化区庞家堡镇市直管镇试点、怀安县左卫镇市特管镇试点，分别下放区、县级审批权限201项和81项。对外开放取得新成绩。招商引资成效明显，全年签订亿元以上项目93项、落地12项，总投资1126.5亿元。与北京的对接合作更加深入，全年与北京签约项目106个，其中71个项目已经落地，到位资金362亿元。多次赴长三角、珠三角招商，签约了一批合作项目。积极参与建立“乌大张”经济合作区。与法国、荷兰、奥地利、以色列等国企业就滑雪装备、畜牧养殖、农产品种植加工等开展了合作。与德国能源署就新能源的开发利用签署了正式合同。投资2亿美元的辛普劳马铃薯深加工项目正在实施。全民创业深入推进。全市融资性担保机构发展到28家，为600多户企业担保贷款20多亿元。张家口市高层次人才创业园挂牌启动，成为全省首家“环首都高层次创业园”。建成大学生创业园3家，入驻大学生创业团队169个，带动就业1100人。全年新增各类市场主体30370个。市中小企业公共服务平台被工信部认定为“第四批国家中小企业公共服务示范平台”。

(五)民生事业持续改善。年初确定的十件民生实事均已完成年度任务，民生领域支出占到公共财政预算支出的79.5%，同比增加了2.4个百分点。基本保障全面加强。援助就业困难人员3.3万人次，城镇登记失业率控制在4.5%以内。城乡年低保补助标准分别提高到2880元和1560元。解决了39.6万人饮水安全问题。新增县级公立医院综合改革试点15家，全部实施了药品零差率销售。新农合参合率97.2%，筹资标准提高到390元。销售惠民蔬菜2万多吨，直接让利于民978万元。新开工建设保障性住房和棚户区改造住房14110套，竣工10237套，分配入住9357套。既有建筑热计量改造完成76万平米，液化气、压缩天然气改造完成4232户。建设农村公路198.6公里，改造县乡公路350公里，改造中心城区小街巷36条，改造及新建公厕40座。19.35万贫困人口稳定脱贫，崇礼县率先实现脱贫出列。各项事业加快发展侯卫东官场笔记。主城区中小学标准化建设扎实推进，二十中改扩建、高新二小新建等6个项目开工建设。出台了《张家口市振兴高中教育大纲》，高考成绩再创历史新高，本科一批、二批上线率首次超过全省平均水平。宣化区、下花园区、万全县通过国家义务教育发展基本均衡评估验收。市职教中心开设“3+4”本科直通班。市特殊教育学校荣获“全国教育系统先进集体”称号。市档案馆、博物馆、图书馆新馆开工建设。察哈尔都统署旧址修缮、大境门加固工程顺利完工。蔚县剪纸文化产业园区、涿鹿三祖文化产业园区、张北中都草原集聚区被评为我省首批文化产业试验园区。14件文艺作品获第十一届“燕赵群星奖”，获奖总数全省第一。成功承办了环北京职业公路自行车赛、“奥林匹克日”长跑活动、康保草原国际马拉松等大型体育赛事。实施了县级医院、农村急救体系、乡镇卫生院等90个卫生基础设施建设项目。完成20个国医堂、100个中医药特色示范村卫生室和3个中医药特色示范社区卫生服务站建设。“单独两孩”政策有序实施。社会秩序安定和谐。6599家持证餐饮单位完成了量化分级评定，占持证餐饮单位总数的93.8%。5家药品生产企业和12家批发企业分别通过新版药品生产、经营质量管理规范认证。我市被列为全国食品安全城市创建试点，张北、怀来等8个县区实施了食品药品安全县(区)创建活动。平安张家口建设深入推进，严厉打击了各类违法犯罪行为。下大力做好安全生产工作，加大煤矿整合关闭、尾矿库专项整治等工作力度。集中解决了一批疑难信访案件和群众反映的实际问题，和谐稳定的发展局面进一步巩固。

过去的一年，我们自觉接受市人大、市政协的监督，广泛听取各民主党派、工商联和无党派人士意见建议，认真办理人大代表建议和政协委员提案。深入开展党的群众路线教育实践活动，切实改进作风，严格执行“八项规定”和“约法三章”，全面清理超标公务用车和办公用房，政府系统的全市性会议和发文同比分别下降30%和20%，“三公经费”同比下降12.3%。认真落实中央和省委巡视组以及国办督查组的整改意见，加大廉政建设和反腐败力度，服务型政府建设取得新进展。双拥共建、国防动员、民政慈善、民族宗教、外事侨务、妇女儿童、防震减灾、物价、审计、监察、人防、统计、工商、质监、商检、气象、供销、档案、地方志、残疾人事业等工作都取得了新的成绩。

各位代表!过去的一年，我市经济社会发展经受了严峻考验，困难和挑战高于预期，成绩来之不易。在此，我代表市人民政府，向在各条战线和各个岗位上辛勤劳动、无私奉献的广大干部群众，向给予政府工作热情支持的人大代表和政协委员，向各民主党派、工商联、各人民团体和社会各界人士，向驻张部队指战员、武警官兵和政法干警，向各位老领导、老同志，向在外地工作的张家口籍同乡，向所有关心支持张家口建设的海内外朋友，致以崇高的敬意和衷心的感谢!

各位代表!在肯定成绩的同时，我们也清醒地看到，在调结构、转方式、促发展过程中，我市还面临着不少问题，三次产业结构不尽合理，农业大、工业小、三产水平低的问题比较突出，传统产业仍居主导地位，新兴产业尚未形成有力支撑；城镇化发展水平较低，园区数量、规模和档次不大不高；创新驱动能力不足，发展方式较为粗放；政府债务沉重，县域经济薄弱，城乡居民收入较低，农村贫困人口众多；受土地、资金、手续办理难度加大等因素的制约，“三馆”建设、主城区教育布局调整等民生实事进度还不够快。全市地区生产总值、固定资产投资、全部财政收入、社会消费品零售总额、规上工业增加值5项指标没有完成预期目标。二、2015年总体要求和工作安排

今年是全面深化改革的关键之年，是全面推进依法治国的开局之年，也是全面完成“十二五”规划的收官之年，更是申办冬奥会的决胜之年。我们要牢牢把握京津冀协同发展和申办冬奥会两大历史机遇，按照省委提出的“脱贫致富”和“生态涵养”两大任务，全面落实市委十届六次全会精神，紧紧围绕“四型经济”重大发展战略，按照“适应经济新常态，激发增长新动力，彰显实干新风貌，开创发展新局面”的主题，苦干实干，坚持不懈，推动张家口经济社会发展不断取得新成效。

2015年政府工作的总体要求是：全面贯彻党的十八大和十八届三中、四中全会精神，认真落实中央和省经济工作会议、省委八届九次全会和市委十届六次全会重大部署，全面发展“四型经济”，力争冬奥会申办成功、京津冀协同发展取得重大进展，统筹做好调结构、稳增长、促改革、优环境、惠民生各项工作，加快张家口绿色崛起进程。

2015年主要预期目标是：全市生产总值增长7%；固定资产投资增长17%；全部财政收入增长6.5%，其中地方公共财政预算收入增长8%；社会消费品零售总额增长11%；城乡居民人均可支配收入分别增长10%和11%；实际利用外资和进出口均增长8%；规模以上工业增加值增长6%；单位GDP能耗下降3%，化学需氧量、二氧化硫、氨氮、氮氧化物排放量分别削减0.5%、0.5%、2.5%、1.4%；城镇登记失业率控制在4.5%以内；居民消费价格指数控制在3%左右；15万贫困人口稳定脱贫。

做好今年的各项工作，必须以市委十届六次全会确定的“四型经济”发展纲要和发展规划为统领，按照纲要确定的目标、任务和举措，切实加大落实力度，积极推动规划实施，统筹推进“四型经济”的融合、协调、创新发展，真正使其成为提升发展速度的新引擎、加速转型发展的“操控室”、实现绿色崛起的“驱动器”。

2015年，重点做好以下工作：

(一)全力以赴争取申奥成功。扎实做好迎评准备工作。把3月份迎接国际奥委会考察作为重中之重，配合北京精心做好每一项工作。继续推进赛场核心区周边、主要交通廊道和可视面造林绿化，以及环境整治工作，加快赛场区域道路互通等基础建设，推动京张高铁崇礼支线、京北一级公路等项目取得突破性进展，分批启动列入申奥规划的项目，为下一步工作打好基础。加快发展冰雪产业。以发展冬奥文化旅游为重点，启动实施京张文化体育旅游带、奥林匹克冰雪文化谷等项目。办好冰雪产业园区，力争尽快引进一批雪具、雪服和雪上装备等生产企业，延长滑雪产业链条。开展以申奥为主题的冬季旅游惠民、冰雪嘉年华等活动。广泛开展冰雪运动进校园、进公园、进万家等活动，普及群众性冰雪体育运动，特别是让更多的青少年喜爱和参与冬季冰雪运动。推动申奥宣传持续升温，打造开放、文明的城市和人文形象，营造全民支持申奥、参与申奥的浓厚氛围。

(二)将项目建设作为经济工作的主线。把抓大项目作为重中之重。对重大项目，主要领导必须亲自抓、盯到底。以中小型高科技项目为努力方向，继续巩固发展与上海久有基金和河北国控的合作，积极推进股权基金、PPP等招商引资新模式，大力发展平台经济、分享经济、协作经济，年内力争再引进中小型高科技企业100家以上。加快建设北方硅谷高科新城二期、新材料科技城、西山产业综合体等一批高科技产业综合体，为200家以上高科技企业提供入驻平台。加快沃尔沃发动机、凯悦汽车大部件、张北云计算、察北旗帜乳业、中法生态产业园、卓达新型建材产业园、北京华电中科LED等重大项目建设进度。继续大力推进园区建设。进一步增强园区承载服务功能，集中打造沃尔沃汽车制造园、中瑞中小企业园等一批专业园区。充分利用好省里的支持政策，建设好张江张家口高新技术产业园，将其打造成为我市乃至全省的亮点。各县区至少要建设一个省级园区。进一步深化园区体制的改革和创新，增强园区的活力和竞争力，使“空口袋”尽快立起来。把工业技改作为项目建设的重要任务。市级安排2亿元专项资金，支持企业进行技改升级、转型发展、科技研发、节能减排。深入实施工业“1+1”工程，重点抓好张煤机煤矿废水处理应用、涿鹿东华绿源5万吨无机纤维材料等50项重大技改项目，年内完成工业技改投资315亿元，同比增长15%以上。多渠道、多层次地推动符合条件的优势企业上市融资，年内力争新增上市企业10家。加大“个转企、小升规”的扶持力度，年内力争新增规上企业90家以上。

(三)深入实施创新发展战略。我市是老工业基地，创新发展是根本出路，要以产业创新为核心，不断创造新的经济增长点。以园区为平台，集聚创新型的人才和技术。进一步加强与国内知名科研院所、大专院校的联系，推广政府与科研机构合作、“产学研”一体化的园区建设模式，推进哈工大科技产业园、怀来航天产业园、中关村——张北云计算产业园建设，吸引北京更多的人才、技术、资金进入我市，把北京的科技人才优势变成我市的新兴产业优势，以集聚方式促进全市战略性新兴产业的快速发展。以基金为引导，形成创新型的企业板块。复制和推广与久有基金的合作模式，集中引进具有四代技术的高科技企业，彻底改造我市的产业结构。抓住国家和省设立新兴产业创业投资引导基金的机遇，积极推进与国内外创业投资、股权投资基金的合作，通过完善“创投基金+产业研究+创新服务”三位一体融资服务体系，尽快达到“一个县区一支产业基金”的发展格局。年内将基金规模扩大到10支以上，筹资总额达到50亿元以上。市政府将拿出2亿元设立股权投资基金，引导社会资本重点投向高新技术产业板块，使其尽早成为全市经济发展的主要支撑。立足我市的特殊资源，加快培育和打造优势产业。充分利用我市丰富的风能、光能资源，积极推进“可再生能源应用综合创新示范特区”建设。在争取相关规划获得国务院批准的同时，不等不靠，在就地转化利用方面先行先试。落实好与恒大、英利、亿利资源、光为绿色新能源集团达成的战略协议，尽快促成“特区”建设“从概念到规划，从规划到项目”。利用我市独特的气候、地形条件，打造以崇礼为龙头，覆盖赤城、张北、沽源等周边地区的冰雪运动区域，迅速壮大冰雪产业规模。发挥我市昼夜温差大、土壤无污染、病虫害少的优势，在农业产业化方面搞出一批具有国际标准的品牌。以互联网技术应用为核心，进一步改造提升传统产业。充分运用云计算、大数据和物联网等信息技术，加快4G网络推广应用，实施电子商务和现代物流发展重点工程，推进电子商务应用向供应链上下游协同发展，培育新的业态；引导企业剥离自营物流业务，组建或引进专业化物流公司，发展第三方物流，提高物流信息化、自动化、智能化、精准化水平。同时积极发展新的商业模式。推进信息化与装备制造业的深度融合，鼓励将信息技术广泛应用于产品设计和制造过程，利用信息技术改造提升传统产业生产工艺和流程，加快研发设计与制造工艺系统的综合集成，丰富产品功能，提高产品性能，提高产品的信息技术含量和附加值，提升产品的网络化、智能化水平，推动产品向产业链高端跃升。

(四)加快培育壮大现代服务业。把壮大现代服务业作为优化经济结构的重要途径，力争服务业增加值年均增长13%以上。文化旅游，以建设“中国优秀旅游目的地”为目标，以滑雪旅游为龙头，以推进旅游各要素紧密衔接、延长旅游产业链条为重点，大力发展风景观光、运动体验、人文探索、高端休闲等特色旅游，突出抓好崇礼周边滑雪旅游、草原天路观光、环官厅湖葡萄品游、赤城“关外御温泉城”和金阁山道教文化旅游、蔚县民俗休闲旅游、中华三祖文化旅游、泥河湾国家考古遗址公园等景区建设，形成旅游多点发展、互为补充的格局。金融服务，积极引进国内外大的创业投资、股权投资基金，共同打造产业发展高地。支持企业以发行债券票据、设立产业基金等方式进行直接融资。通过政府引导、社会资本参与的形式引进或组建风险投资、股权投资基金，吸引各类投资机构来我市发展，丰富现代金融业态。创新中小企业金融服务模式，采取“政府搭台、金融机构和企业参与、市场化运作”的办法，搭建完善中小企业政府融资平台，探索“信贷工厂”、“金融超市”等新模式，为中小企业提供综合金融服务。加强社会诚信体系建设，建立健全地方金融监管体制，防范和化解金融风险。科技研发，瞄准北京这个中国最大的人才库，吸引更多高层次人才到我市开展科技研发。激活企业创新主体，鼓励企业加大研发投入、采用先进技术、研发高端产品。加强技术中心建设，重点推进杂交谷子国家工程技术中心、张煤机国家重点实验室和省级拉曼光学工程中心等建设。借助北京创新优势，用足用好国务院推向全国的6项中关村先行先试政策，发挥涿鹿国家级科技孵化器和张家口久远省级科技孵化园的作用，建立科技成果转化服务和交易平台，设立成果转化基金，促进科研合作转化，吸引一批国家级科研院所和高校的科技成果到我市进行转化和产业化。市政府安排科技经费5000万元，对企业获得省级高新技术企业认定、建成国家或省级的工程中心、工程实验室、技术研究中心，以及荣获全国驰名商标、名牌产品的，给予不同数额的奖励。健康养老，适应我国快步进入老龄化社会的实际，充分发挥我市空气良好、交通便利、地热条件突出等优势，借助申奥效应和京张之间联系交流更加紧密等机会，支持社会力量投资兴建各种档次的养老服务机构，同时引进知名医疗机构入驻我市，实现养老和医疗康复的融合发展，打造“健康张家口”养老服务品牌。现代物流，积极发展专业化、社会化的第三方大型物流企业，逐步培育一批生产服务型物流园区和配送中心。做好煤炭物流中心、冀北粮油仓储物流中心等现代物流产业集聚区的标准化、信息化建设，加快京张奥物流产业园区建设步伐，谋划启动空港物流园区建设。电子商务，支持更多企业纳入全省电子商务发展“三年推进计划”，培育一批行业电子商务龙头企业。全面开展电子商务发展“农村火种计划”，加快农村、城市社区电子商务应用推广。

(五)大力提高农业农村发展水平。以规模化、标准化、特色化、集约化为重点，进一步提高农业生产的质量和效益，加快县域经济发展。积极推进土地流转。在尊重农民意愿的前提下，进一步加快土地流转，促进土地向专业公司、家庭农场等新型经营主体集中，加快现代农业发展步伐。年内力争新增家庭农场150户。用抓好工业园区的办法抓好农业园区，一个县至少要有一个现代农业示范园区。抓好塞北国家级现代农业示范区、中荷现代农业园、中芬示范农场等一批农业精品园区建设，推动环首都现代农业示范带建设。加快推进“空心村”的治理和复垦工作。加快推进农业安全性、标准化生产。以建设奥运食品基地为目标，着力推动特色产业“优质安全、标准集约”发展。年内，新认定蔬菜无公害基地15万亩、绿色基地1万亩、有机基地5000亩；新增奶牛标准化规模养殖场40个；建立葡萄酒桑洋河谷产地标准；“张杂谷”发展到40万亩、规模种植示范片区达到10个，多元转化加快推进。大力推进全产业链生产。强化对覆盖一二三产全产业链龙头的扶持，推动农业产业化、工业化、城镇化、信息化的有机融合 落霞小说。依托察北旗帜乳业、塞北现代牧场、宏文牧业等龙头，打造高品质婴幼儿奶粉和高端液态奶产业集群。扶持弘基、雪川、燕北等薯业龙头做大做强，打造国际一流的马铃薯产业基地。加快张北中粮、宣化普甜、康保乾信等一批肉食加工企业建设，打造京津冀肉类食品生产基地。努力推进农民增收。进一步强化农产品产销对接，增加农业经营收益，破解增产不增收难题。加强农民技能培训，培养高素质职业农民队伍。提升农业气象灾害防御能力和水平。继续把扶贫作为一项重大的政治任务坚持不懈推进，加快实施精准扶贫，量身定制扶持办法，精确实施好基础设施改善、富民产业入户等项目。推进赤城“全国光伏扶贫项目试点县”建设，创造扶贫开发新模式。(六)坚持不懈改善生态环境。立足“京津冀水源涵养功能区”定位，向世界一流、奥运标准看齐，朝着“蓝天净水”的目标不断前进。深入治理大气污染。重点推进主城区及县城的集中供热，做好烟煤粉尘治理，基本淘汰或利用清洁能源等技术改造主城区10蒸吨及以下燃煤锅炉，主城区规模以上餐饮企业及单位食堂全部安装油烟高效净化装置。推动洁净煤配送中心建设，在城中村和农村区域积极推广使用洁净型煤，市政供热供电优先使用可再生能源。对宣化、怀来等重点区域采取强有力的措施，务必在大气污染防治方面有根本性改变。对崇礼实行最严格的环保措施，努力实现“近零排放”目标。全面完成主城区11家污染企业搬迁改造，完成7家企业关停淘汰。实施宣钢2台120万吨回转窑脱硫工程等一批减排项目。对年综合能耗5000吨标准煤以上的企业全部纳入监测范围，选择5家重点企业实行能耗在线监测。推进新《环保法》的落实，在全市开展“环境执法建设年”活动，严厉查处环境违法案件。高标准推进造林绿化。重点实施坝上退化林分改造、京冀生态水源保护林等五大工程，完成造林绿化100万亩以上。继续实施好迎宾廊道和赛区绿化及改造工程。特别要采用更加创新、更加有效的市场机制，充分调动造林主体的积极性。大力发展林业产业，建设10万亩林苗一体化工程区和10个千亩林下经济示范园区，实现经济和生态效益的双赢。加大生态环境修复力度。巩固坝上地区节水成果，做好坝下地区推广工作，年内新增节水灌溉面积31万亩。完成水土流失治理面积240平方公里。积极开展矿山地质环境治理恢复，对关闭取缔或转为洞采的矿山、小砖窑等全部进行生态恢复。继续实施好国家、省森林公园、湿地公园保护各项措施。

(七)以国际化标准推动城市发展。围绕建设国际化的“冬奥名城”，全面提升城市建设管理水平，加快建设“清洁、秩序、美丽、文明”的张家口。提高城市规划水平。按照奥运城市的新定位和新要求，以世界发达国家的著名国际化城市作为参照系，瞄准国内先进城市，实施好新一轮城市总体规划的修编工作，搞好城市规划顶层设计，增强城市规划的先进性、科学性和权威性。聘请国内外知名的规划设计公司和世界一流的大师，按照“既体现历史传承、彰显地域特色，又注入国际元素、展示现代风貌”的理念，高标准做好各个功能板块、核心区域、窗口部位、景观节点、地标建筑的规划设计。实行最严格的规划制度，切实维护规划的刚性约束，做到规划一旦确定，不得随意更改，宁可“留白”，绝不“遗憾”。提高城市建设水平。突出精品理念，杜绝低水平建设，城市建设的每一项工程，都要参照国际化城市的标准来进行，城市景点、标志性建筑、广场、街景等都要有鲜明的特色。加快推进世贸一期、怡安街改造等10个城市综合体项目，打造成张家口城市名片。突出低碳绿色，推进可再生能源在建筑上的应用和开发，大力推广使用新型建筑材料。突出做好清水河两侧、城市出入口等重要节点、重点区域的景观提升，使其成为城市中的“明珠”。高标准、大手笔推进开发区建设，率先打造成具有国际化水平的城市发展示范区。启动高铁综合枢纽建设。提高城市管理水平。积极推进与联通、移动等通信公司以及专业化公司的合作，完善城市公共信息服务平台，通过信息技术的广泛应用，打造智慧城市，构建智慧交通、智慧城管、智慧安防等智能化信息系统，提升城市精细化管理水平，使城市变得通畅、整洁、便捷、现代。加快与北京环卫集团合作的对接进度，以北京奥运标准做好城乡环境综合治理。按照“重心下移、职责下沉”的原则，改革城市管理体制，推动管理力量向一线延伸覆盖。整合城市服务资源，建立完善、统一、高效的城管平台，逐步达到“一个热线管到底”。突出抓好环境容貌综合整治，塑造优美的城市形象。将创建全国文明市县与申办冬奥会结合起来，深入开展文明城区、文明社区、文明单位创建和“爱我城市、告别陋习”等主题活动，促进广大市民提高文明素质，养成良好的行为规范。

(八)进一步扩大改革开放。全面落实各项改革的总体部署，推动重要领域改革取得新进展，大力提升开放型经济水平。继续简政放权。继续清理取消行政审批事项、流程和收费项目，承接好国务院和省下放的审批事项，重点清理规范前置审批条件，对保留的行政审批事项进行规范管理。完善行政审批服务中心体制，逐步实现“一个印章管到底”。深化各项改革。深化农村综合改革，年内完成705万亩土地的承包经营权确权登记颁证工作，完善张北土地流转收益保证贷款、万全新型经营主体试点，在张北、尚义两县探索建立农村产权交易平台。扎实推进供销社综合改革。继续深化医药卫生体制改革，巩固28个县级公立医院改革成果。推动庞家堡镇正式按直管体制运行。完成机关公车制度改革。深化水、电、气等资源性产品价格改革。扩大开放合作。围绕京津冀协同发展规划，全面落实好京冀“6+1”和津冀“4+1”合作文件确定的各项目标任务。进一步加强与在京的企业总部、院所、高校的对接合作，推动京张两地更深层面、更广领域的协同发展。巩固和发展与“长三角”、“珠三角”合作成果，开拓与欧洲国家的经贸合作。加大招商引资工作在年度领导班子考核中的权重，增加对县区当年签约、落地项目和到位资金的考核。建立代理招商、驻点招商等工作评估机制，充分发挥各种招商中介平台作用，吸引更多的国内外客商到我市考察投资。在开发区和相关部门，建立若干招商团队，形成有效的竞争机制。强化创业服务。认真落实国家和省、市支持民营经济、全民创业的政策措施，年内力争每个县区建成一处创业园或孵化基地，为300个创业团队提供创业平台。重点加快桥东区等5个县区创业基地、创业园建设，全市改建、新建厂房面积10万平米。进一步加强市级中小企业公共服务平台建设，登记入驻企业和中介机构争取达到5000家。年内力争新增市场主体1.5万个。

(九)持续有力改善民生。按照保基本、兜底线、促公平的要求，切实解决好事关群众切身利益的问题。全力扩大就业。统筹解决好高校毕业生、城镇就业困难人员等重点人群的就业问题。强化保障体系。突出对基本民生与低收入群众生活的保障，企业退休人员养老金标准再提高10%以上。密切关注居民消费价格水平变化，落实社会救助和保障标准与物价上涨挂钩联动机制。促进教育公平。继续下大力推进义务教育均衡发展，认真做好迎接国家、省评估认定工作。实施第二期学前教育三年行动计划，加快振兴高中教育，推动普高课程和综合素质评价改革，大力发展职业教育，努力构建现代职教体系。进一步提高市属高校和特教办学水平。继续实施好“蛋奶工程”和营养改善计划。提升医疗保障。实施230个基层卫生项目，加快推进张家口肿瘤医院、天泽妇女儿童医院等项目建设，强化重大疾病防控，建立健全医疗纠纷第三方调解机制。新农合参合率稳定在95%以上，筹资标准提高到490元。推进“计生惠民”政策进一步落实。实施文体惠民。打造《魂系京张》、《大好河山》等一批文化艺术精品。做好察哈尔都统署、察哈尔抗日同盟军两大展览。编制实施《张家口文化产业整体提升规划》和《长城保护开发规划》。谋划市奥体中心项目。推进学校、企业体育设施向社会开放。完善公用设施。实施一批城市主干道、小街小巷新建改建工程，加快垃圾污水处理、水源地改造等工程进度。推进平安城市建设。始终保持对各类犯罪行为的高压态势，严厉打击涉毒、盗抢等各类犯罪。建立市县乡村“四位一体”食品药品监管机制，深入开展食品安全试点城市创建和食药安全县(区)创建工作。加大安全生产工作力度，确保不发生重大安全生产事故。搞好网格化社会管理试点。着力解决信访突出问题，依法维护信访秩序。

各位代表!虽然我市财政收支矛盾突出，但我们要确保民生投入只增不减，扎扎实实办好十件民生实事。一是实施中心城区义务教育学校标准化提升工程。建国路小学、南小区小学年内投入使用，二十中、大境门南区小学、主城区南部小学、新区二小4个已开工项目主体完工，加快推进建院改建四中、十中改建初中、新建建院小学、新区中学综合楼4个项目建设，力争盛华小学、新区三小开工。二是完善城市功能。解决城市道路交通瓶颈，实现东环路至北环路连接线贯通。继续推进“三馆”建设，年内市档案馆、博物馆、图书馆实现主体完工。三是提高城市绿化水平。启动5000亩的吉家房森林公园建设工程，新建10个城市公园游园，打造城市绿肺。四是积极扩大就业规模。年内实现城镇新增就业4.5万人、农村劳动力转移就业3.5万人。五是增强住房保障能力。新开工各类保障性住房和棚户区改造住房1.2万套，完成农村危房改造2万户。六是健全养老服务体系。居家养老服务中心覆盖率达到60%，农村互助幸福院覆盖率达到65%。提高居家养老呼叫网络平台服务水平，年内入网人数超过3万人。七是解决农村人口饮水安全问题。年内实现24.52万农村人口饮水安全，让更多的农村居民喝上放心水。八是做好惠民蔬菜工作。全年直接让利于民1000万元以上，2015年春节肉菜补贴215万元。九是确保人民群众饮食用药安全。全面实施“明厨亮灶”工程，主城区持证餐饮服务单位达到70%以上，推动药品零售经营企业规模化、规范化发展，药品零售连锁率达到65%以上。十是开展好旅游惠民活动。推出4条旅游惠民线路，开通旅游惠民直通车，做好旅游惠民“一卡通”的发售工作。我们一定把保障和改善民生作为政府的根本职责，让改革发展的成果更多更公平地惠及全市人民!

三、进一步加强政府自身建设

面对新形势新任务，我们必须时刻牢记职责和使命，全面推进法治政府、效能政府、廉洁政府建设，着力提升政府形象，让人民更加满意。

(一)进一步提升政府工作的法治化水平。认真贯彻落实党的十八届四中全会决定，把依法行政贯穿到政府工作的各个领域、各个层面、各个环节，严格做到法无授权不可为。市政府的重要会议和重大决策，严格履行“公众参与、专家论证、风险评估、合法性审查、集体讨论决定”等法定程序。建立重大决策终身责任追究制度及责任倒查机制，对违反决策程序、出现重大决策失误的严格追究相关责任人的法律责任。加快推进执法重心和执法力量向县区下移，合理配置执法力量，减少执法队伍种类，逐步推进城市管理、环境保护、安全生产等重点领域综合执法，着力解决权责交叉、多头执法等问题。严格实行行政执法人员持证上岗和资格管理制度，严格规范行政执法机关及执法人员行政执法行为，严格执行重大行政许可、行政处罚等行政执法行为法制审核制度。加强行政复议工作，畅通行政救济渠道。自觉接受人大的法律监督、工作监督和政协的民主监督，加强协商议政。保障和支持审计、监察等部门依法独立行使监督权。

(二)深入推进政府系统党风廉政建设和反腐败工作。加强政府系统惩防体系建设，严格落实重大事项报告、述职述廉和责任追究制度。严厉查处权力寻租、损害群众利益等行为，坚决查处违法违纪案件，对腐败分子坚决依法严惩。严肃查处工程建设、土地出让、政府采购等领域的腐败行为。结合新一轮政府机构改革，按照“职权法定、权责一致”的原则，加快建立政府权力清单、责任清单和负面清单制度，消除权力寻租空间。深入落实中央“八项规定”和国务院“约法三章”，坚决纠正“四风”问题。厉行节约反对浪费，严格控制一般性支出，确保“三公”经费只减不增。全面推进财政预算决算、公共资源交易、民生和公共服务等领域的政府信息公开。

(三)切实改进工作作风，提高各级政府的执行力和行动力。巩固党的群众路线教育实践活动成果，把更多的精力放在主动作为、狠抓落实上。各级政府及政府各部门对法定职责范围内的行政事务，要勇于担当、敢于负责，执行落实拒绝理由。坚决纠正不作为、乱作为，坚决克服慵懒散慢，坚决惩处失职渎职。结合行政审批权力的不断下放，积极推进管理向治理的转变，把工作重心放在市场监管和公共服务上。进一步健全抓落实的制度，对已经确定的各项工作，层层分解，落实到岗，责任到人，贯通决策部署的“最先一公里”和工作落实的“最后一公里”，把市委的决策和群众的要求变成各级政府加快发展的具体行动。

各位代表，新的一年，我们的任务艰巨而繁重，使命重大而光荣。让我们在省委、省政府和市委的正确领导下，在市人大、市政协的监督支持下，抢抓机遇，真抓实干，在新常态下创造新的更大的业绩!
